# Imaging metastatic bone disease from carcinoma of the prostate

**DOI:** 10.1038/sj.bjc.6605334

**Published:** 2009-09-29

**Authors:** C Messiou, G Cook, N M deSouza

**Affiliations:** 1 Cancer Research UK Clinical Magnetic Resonance Research Group, Institute of Cancer Research and Royal Marsden NHS Foundation Trust, Downs Road, Surrey SM2 5PT, UK; 2 Department of Nuclear Medicine, Royal Marsden NHS Foundation Trust, Downs Road, Surrey SM2 5PT, UK

**Keywords:** prostatic neoplasms, neoplasm metastasis, diagnostic imaging

## Abstract

Imaging bone metastases from prostate cancer presents several challenges. The lesions are usually sclerotic and appear late on the conventional X-ray. Bone scintigraphy is the mainstay of lesion detection, but is often not suitable for assessment of treatment response, particularly because of a ‘flare’ phenomenon after therapy. Magnetic resonance imaging is increasingly used in assessment, and newer techniques allow quantitation. In addition to ^18^F-fluorodeoxyglucose (^18^FDG), newer PET isotopes are also showing promise in lesion detection and response assessment. This article reviews the available imaging modalities for evaluating prostatic bony metastases, and links them to the underlying pathological changes within bone lesions.

Prostate cancer is the second most common cancer in men, accounting for 1 in 9 of all new cancers, and with more than 670 000 new diagnoses annually worldwide. The metastatic spread is primarily in the skeleton (supporting the ‘seed-and-soil’ hypothesis described by Paget in 1889) in which lesions are often located in vertebra and ribs because of dissemination through Batson's venous plexus. The spread in bone also follows the distribution of adult red bone marrow, that is, skull, thorax, pelvis, spine, proximal long bones (Imbriaco *et al*, 1998; Scher, 2003), subsequently progressing to involve adjacent cortical bone. Preclinical models confirm that skeletal sites rich in cellular marrow with active turnover show increased cancer localisation (Schneider *et al*, 2005). Although predominantly osteoblastic, osteoclast activation also has an important role in the growth of sclerotic metastases in the bone. In a study of 68 men with prostatic bone metastases who underwent surgery for stabilisation of pathological fracture or impending fracture, most metastases were osteoblastic, but 29.1% had metastases that were osteolytic or mixed (Cheville *et al*, 2002).

Skeletal metastases occur in approximately 90% of patients presenting with advanced prostate cancer, and the burden of bone disease directly correlates with survival (Cooper *et al*, 2003, Carlin and Andriole, 2000). After treatment of the primary site, bone is the first site of relapse in more than 80% of cases (Clamp *et al*, 2004). Plain film and bone scintigraphy studies form the mainstay of detection, but they underestimate true incidence. In one autopsy series of 1589 men with prostate cancer (47% were unsuspected), the incidence of metastatic bone disease was 90% (Bubendorf *et al*, 2000).

The detection of bone metastases indicates progression to lethal prostate carcinoma (Scher, 2003). At this stage, complete remissions are rare and onset of the complications of bone metastases are likely (Clamp *et al*, 2004). The investigation of therapeutic interventions to slow the progression of bone disease and its complications make the need for accurate assessment of disease burden in the bone and its response to treatment of fundamental importance. PSA is used widely to monitor response to therapy, with a decrease in PSA to the normal range after treatment used as a predictor of prolonged response in many patients (Ruckle *et al*, 1994). However, PSA levels are influenced by both soft tissue and bony disease and PSA does not always correlate with tumour burden.

Imaging bone disease in prostate carcinoma frequently involves a cascade of studies that start with Tc^99m^ methylene diphosphonate (Tc^99m^MDP) bone scintigraphy, backed up by plain film correlation and followed by magnetic resonance imaging (MRI), computerised tomography (CT) or even positron emission tomography (PET)/CT. The implications of this multistep approach involve patient time, imaging time, costs and radiation dose. Validation of imaging biomarkers for bone derived from these studies has been hindered by a lack of a gold standard, as histological verification is not appropriate. Previous arguments that MRI is too costly and time consuming need to be revisited, particularly in the setting of its increased availability, and with the development of functional imaging approaches. Currently, the assessment of therapeutic response in clinical trials relies solely on qualitative assessment on bone scintigraphy, as Response Evaluation Criteria In Solid Tumours (RECIST) criteria classify osteoblastic bone metastases as non measurable (Eisenhauer *et al*, 2009). This article reviews the characteristics of prostate bone metastases recognised with various imaging techniques in the context of their pathogenesis and explores the potential of these techniques for assessing tumour burden and response to therapy.

## Bone scintigraphy in assessment of bone metastases

**Figure 1:**
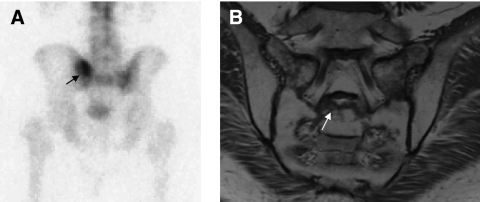
False-positive MDP bone scintigraphy. A male patient with prostate cancer and left sided sacral pain previously treated with IMRT, PSA <0.04. MDP bone scintigraphy (posterior view, **A**) showed a solitary focal area of uptake in the left side of the sacrum (arrow), interpreted as a bone metastasis. Symptom progression with bilateral sacral pain but PSA remaining <0.04 prompted an MRI, which showed bilateral oedema in the sacral ala (T1W coronal), (**B**) and a fracture through S2 (arrow) but no evidence of metastasis.

The popularity of bone scintigraphy arose from its comparisons with plain film radiography. Bone scintigraphy can detect a 10% change in bone mineral turnover, whereas the bone must demineralise by 50% before a lesion is detected by plain film. It can also detect bone metastases up to 18 months before plain film reveals them (Taoka *et al*, 2001). However, because bone scintigraphy images the secondary effects of the tumour on the skeleton, false positives occur from degenerative change, inflammation, Paget's disease and trauma (Figure 1). The osteoblastic response that occurs as a result of bone healing/flare response can also lead to a false-positive diagnosis of disease progression. The sensitivities and specificities for detection of bone metastases by MDP bone scintigraphy have sometimes been quoted, but the absence of a histological gold standard means that these are not sensitivities and specificities in the true sense. Comparators vary from study to study, but PSA, soft tissue disease, follow-up and other imaging modalities are often used as a gold standard, all with their own limitations.

**Figure 2:**
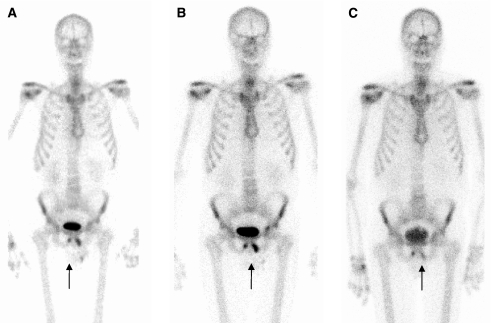
Flare response on MDP bone scintigraphy: metastatic disease in the inferior pubic rami (arrows, **A**) showed increased uptake 3 months after chemotherapy (**B**) that diminished at 6 months (**C**).

The flare phenomenon on radionuclide bone scan in patients with prostate cancer has been reported at anywhere between 6 and 25% and is also a feature observed on plain film. It may be because of an increase in blood flow caused by an inflammatory response or an increased turnover of hydroxyapatite in the new bone laid down as part of the healing process. In prostate cancer, if the scan taken 3 months after introduction of therapy shows worsening of disease, there is a high probability that this is real. If, however, the patients' clinical parameters indicate a response, then flare should be considered. A follow-up scan at 6 months can resolve the issue (Figure 2) (Levenson *et al*, 1983; Pollen *et al*, 1984).

Regardless of the flare phenomenon, the sensitivity of bone scintigraphy in detecting a response to therapy remains questionable; metastases showed by bone scintigraphy have been shown to remain stable despite other parameters indicating a response (Scher, 2003). Coombes *et al* (1983) found purely sclerotic bone metastases impossible to assess on bone scintigraphy, as increased sclerosis without scintigraphic changes occurred in the responding and non-responding patients. In the responding patients (as judged by disease in non-osseous sites), any detectable response on bone scan is often delayed by up to 6–8 months and it can take over 2 years for complete resolution of bone scintigraphy findings (Scher, 2003), even when all of the disease has been eliminated from the bone. Conversely, a stable positive scintigraphic lesion, in conjunction with a fading sclerotic lesion on radiographs in a positive scintigraphic lesion, can be a sign of progression (Pollen *et al*, 1984). A further source of debate is the occurrence of a new lesion on bone scintigraphy. Previously, this was thought to rule out flare response but it has been shown that appearance of a new lesion on bone scans or plain film within 6 months of initiation of therapy can be a part of the flare response as a result of the healing of previously occult lesions (Ciray *et al*, 2005).

Descriptive reports provided by bone scintigraphy, although useful for diagnosis, are limited when assessing the response to therapy, in which more quantitative information is desirable. The Bone Scan Index proposed by Imbriaco *et al*, which quantifies the proportion of the skeleton involved by tumour as well as the distribution of disease, has not been widely adopted. Other proposals include an automated assessment of the percentage of involvement by metastatic bone disease on bone scintigraphy to monitor response to therapy. Although scoring systems of this type may relate to prognosis and response to therapy, they can be time consuming and variable (Sabbatini *et al*, 1999; Noguchi *et al*, 2003). Other limiting factors are the lack of anatomical detail. Combined single-photon emission computerised tomography (SPECT) and X-ray CT improve anatomical detail and reduce the number of equivocal lesions detected on bone scintigraphy (Even-Sapir *et al*, 2006). Bone scintigraphy therefore does have a role in the assessment of prostatic bone metastases but should not be used in isolation when considering response to therapy.

## MRI for assessment of bone metastases

Magnetic resonance imaging is potentially the technique of choice in evaluating prostate bone metastases as it is sensitive to early changes in bone marrow that precede the osteoblastic response in the bone matrix. Metastasis to bone marrow leads to a lengthened T1 relaxation time and signal loss, which contrasts with the surrounding high signal marrow fat. The conspicuity of bone metastases can sometimes be increased by T2-weighted fat-suppressed sequences such as short tau inversion recovery (STIR).

**Figure 3:**
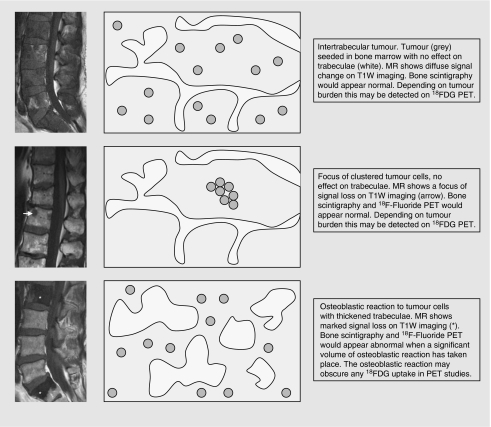
Schematic describing the relationship between patterns of tumour seeding in bone marrow and imaging findings.

Magnetic resonance has been shown to detect bone metastases in 37.5% of patients with negative or inconclusive bone scan and plain films, and one prospective study indicates sensitivities and specificities of 100 and 88% for MRI and 46 and 32% for bone scintigraphy (Lecouvet *et al*, 2007). The discrepancy between these modalities arises because even with extensive marrow involvement by metastases, the amount of bony matrix destroyed is small (Taoka *et al*, 2001). In particular, vertebral bodies have a large medullary cavity, and hence the cortical involvement leading to positive bone scintigraphy occurs late (Taoka *et al*, 2001). Furthermore, tumour cells may reside between trabeculae in which they may be recognised on MR but not on bone scintigraphy or plain film (Figure 3) (Yamaguchi *et al*, 1996). In one study, all intramedullary lesions on MRI were negative on bone scintigraphy regardless of size; once there was a cortical involvement, bone scans were likely to be positive. Positive bone scan findings were always associated with MRI evidence of cortical involvement. In addition, transcortical lesions had a higher incidence of positive bone scan findings than subcortical lesions in which lesion detection was shown to be size dependant (Taoka *et al*, 2001). Although the comparisons have been carried out without SPECT, it is unlikely to have an affect on those cases in which disease is confined to the medullary cavity. Interobserver agreement is also greater in MR studies than in bone scintigraphy (Algra *et al*, 1991).

The RECIST criteria applied to MRI of the axial skeleton in one small study have confirmed the superior sensitivity of MRI to bone scintigraphy in the detection of bone metastases and have shown that it may have a role in quantitatively following bone metastases. Despite classifying diffuse bone involvement as non-measurable because it was impossible to obtain longest axis dimensions, this study increased the number of patients with ‘measurable’ metastatic lesions by 29% (Tombal *et al*, 2005).

Although conventional MRI lacks whole-body coverage, it is possible to cover the whole spine and pelvis (in which the majority of prostate cancer metastases arise) in minutes and to include the femoral necks that are at risk of pathological fracture. A study of 66 patients with high-risk prostate cancer has shown no cases of isolated peripheral metastases (Lecouvet *et al*, 2007). With newer scanners, coverage of the whole skeleton with MRI by use of whole-body coil arrays or moving table arrangements is possible but it is time consuming, and the difficulty with MR interpretation of certain areas, such as ribs, does not make such intensive imaging worthwhile.

**Figure 4:**
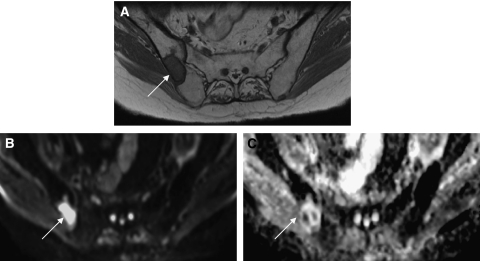
A male patient with prostate cancer metastases to bone. T1W axial MRI pelvis (**A**) shows a metastasis within the right iliac bone (arrow). High signal within the lesion on the diffusion-weighted MRI of the pelvis (**B**) indicates that diffusion within the metastasis is less restricted than diffusion in the surrounding normal marrow. An apparent diffusion coefficient (ADC) map of pelvis (**C**) generated from the diffusion-weighted imaging data (**B** values 0, 50, 100, 250 500, and 750) provides a quantitative index of water diffusion within the tumour. The ADC map also shows heterogeneity of water diffusion within the tumour not shown by conventional T1W imaging.

**Figure 5:**
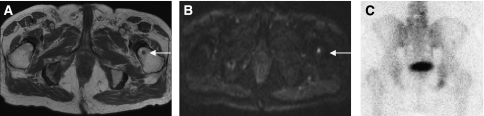
Comparison of MRI and MDP bone scintigraphy: pelvic T1W MRI (**A**) and b750 DWI (**B**) of a patient with carcinoma of the prostate shows a new small metastasis (arrows) involving the left neck of femur. This small intramedullary lesion has not evoked enough osteoblastic reaction to become visible on bone scintigraphy (**C**).

Newer MRI methods are addressing the lack of quantitative assessment of bone metastases. Dynamic contrast-enhanced MRI provides information on the perfusion and permeability of tumours and has shown potential in detecting metastatic bone disease and monitoring response to therapy (Montemurro *et al*, 2004). Diffusion-weighted imaging (DWI) can detect differences in water diffusion between tissues, which can be measured as apparent diffusion coefficients (ADC). Thus, DWI can be used to record the restriction of water in and around tumour cells and changes in water diffusion that occur as a result of changes in cellular density and membrane integrity after therapy (Charles-Edwards and deSouza, 2006). Although DWI has been validated in several soft tissue tumours (Montemurro *et al*, 2004), its use in metastatic bone disease is in its infancy. In fact, mature bone marrow is particularly amenable to interrogation with DWI, because its high fat content results in a significant diffusion restriction. When diffusion-restricted fatty marrow is replaced with water containing tumour cells, these foci become conspicuous as areas of increased diffusivity. This generates excellent contrast between normal marrow and tumour. The derived ADC maps allow quantitative assessment of changes in cellularity in response to therapy (Figure 4). Furthermore, recent hardware and software advances have led to whole-body DWI assessment of the skeleton in very reasonable timeframes, and anatomic images can be fused to allow assessment of threat to spinal cord or nerve roots. Preliminary data suggest that DWI may surpass conventional T1W imaging and STIR for lesion detection (Figure 5) and is equally as effective as ^11^C choline (Luboldt *et al*, 2008).

Magnetic resonance spectroscopy has also been used to differentiate malignant from benign bone tumours. Interrogating choline metabolites on ^1^H MRS or phosphomonoesters on ^31^P MR spectroscopy in small studies has shown some success (Sijens *et al*, 1997; Wang *et al*, 2004). However, both techniques are limited to large and relatively superficial lesions and are extremely time intensive and have limited anatomical coverage.

## PET for assessment of bone metastases

The limitations of bone scintigraphy have spurred an interest in PET imaging in prostatic bone metastases. ^18^F-fluorodeoxyglucose (^18^FDG) is a non-specific tracer. It is an analogue of glucose, thereby reflecting metabolism and detecting the increased glucose transport and glycolysis associated with several tumour types. It gains entry into cells by membrane transporter proteins such as Glut1, which are expressed in many tumours. It is phosphorylated intracellularly to FDG-6-phosphate and retained within malignant cells. Tumour hypoxia may also increase ^18^FDG accumulation through activation of glycolysis. The quantitative parameter used is typically a standardised uptake value (SUV), which represents the tissue activity within a region of interest corrected for the injected activity and for the patients' body weight. Sclerotic metastases show little ^18^FDG uptake compared with lytic lesions (Fogelman *et al*, 2005). The exact cause for this reduced uptake is not known but speculation centres on lower volumes of tumour associated with sclerotic metastases, a difference in sclerotic tissue metabolism or attenuation of photons by densely calcified tissues. ^18^FDG PET is therefore less sensitive than MDP bone scintigraphy in the identification of sclerotic metastases (Shreve *et al*, 1996) and had been shown to detect only 18% of sites seen on bone scintigraphy in patients with stable or responding disease. The lesions not detected by ^18^FDG PET are often those that are stable on follow-up bone scintigraphy (Morris *et al*, 2002). However, some lesions detected on ^18^FDG PET alone become positive on bone scintigraphy after some time. It is likely therefore that ^18^FDG PET is detecting active disease within bone marrow before a significant secondary bone reaction or cortical involvement. A new lesion or a rise in SUV within a lesion that is correlated with a rise in PSA indicates disease progression, and Morris *et al* (2005) have shown that ^18^FDG PET is very promising as an outcome measure for prostate cancer.

Even without CT correlation, ^18^FDG PET offers superior resolution to conventional gamma camera imaging, and the acquisition of tomographic images is routine. The combination with CT on hybrid PET/CT scanners offers the advantage of fusing structural and functional data. The concordant lesions found on both PET and CT are highly likely to represent bone metastases; however, this likelihood falls if only the PET is positive and is reduced even further if the lesion is solitary. Solitary lesions positive on PET but not on CT should be interpreted with caution. Lesions seen on CT but not on ^18^FDG PET have an even lower positive predictive value (Taira *et al*, 2007).

**Figure 6:**
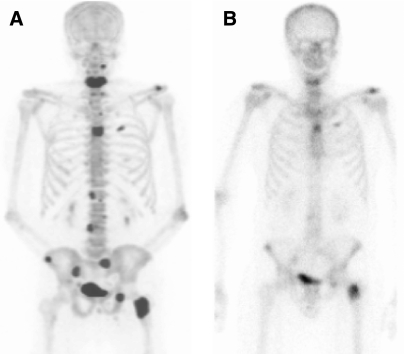
Comparison of ^18^F-fluoride PET and MDP bone scintigraphy. (**A**) ^18^F-fluoride shows an increased number of metastatic deposits and better resolution than MDP bone scintigraphy (**B**) on the same patient.

Another non-specific bone PET tracer ^18^F-fluoride (half-life 110 min) is taken up by bone metastases and related to their osteoblastic activity. Diffusion of ^18^F-fluoride through capillaries into bone leads to fluoroapatite formation. This is stored on the bone surface in which turnover is the greatest. As the first pass extraction of ^18^F into bone is almost 100% (compared with 64% for the larger phosphate complexes used in traditional bone scintigraphy), ^18^F-fluoride PET shows a high contrast between normal and abnormal bone (Figure 6) (Langsteger *et al*, 2006). It is thus an attractive imaging agent with high-quality images that are likely to be more sensitive than conventional bone scintigraphy for detecting both osteoblastic and osteolytic bone metastases. The earlier detection of osteoblastic activity by ^18^F-fluoride PET than with MDP bone scintigraphy (Even-Sapir *et al*, 2006) is further enhanced by the increased spatial resolution of PET imaging that increases the sensitivity of the technique. Specificity problems with ^18^F-fluoride PET are similar to those encountered with traditional MDP bone scintigraphy, although the improved spatial resolution and the combination with CT helps to differentiate between benign and malignant processes in equivocal cases by clarifying the exact anatomical location of a lesion within a bone, for example, vertebral body or end plate (Even-Sapir *et al*, 2006). A combined ^18^F-fluoride and FDG PET examination has been proposed by some researchers (Hara *et al*, 1998), offering the attractive prospect of additive information from osteoblastic and metabolic activity.

Other PET tracers currently under evaluation include ^11^C methionine and ^11^C/^18^F-choline and ^11^C-acetate derivatives. The interest in choline has grown from evidence that it is transported into cells, phosphorylated and thus trapped within cells and used for the synthesis of phospholipids. Malignant cells have elevated levels of choline and upregulation of choline kinase activity as a result of increased cell turnover. ^18^F-choline PET/CT has shown the potential to both upstage and downstage bone disease in prostate cancer when compared with Tc^99m^MDP bone scintigraphy. It has a longer half-life (110 min) with slightly better imaging quality than ^11^C-choline (half-life 20 min) imaging but excretion into urine interferes with pelvic imaging. As with ^18^F-FDG, there is some evidence to suggest reduced uptake in patients treated with antiandrogen therapy (Langsteger *et al*, 2006). ^11^C-choline PET may have other advantages over ^18^F-FDG PET for detection of pelvic disease and bone metastases. Pelvic imaging is made easier as its urinary excretion is negligible. However, ^11^C-choline does accumulate in liver, kidney, spleen and pancreas, making assessment of the upper abdomen difficult (Hara *et al*, 1998). The short half-life of 20 min limits its use to sites with cyclotrons. Most studies of choline in prostate cancer have interrogated localised or nodal disease. In one retrospective analysis, ^11^C-choline PET/CT was valuable in the assessment of metastatic bone disease in terms of detection, localisation and characterisation (Tuncel *et al*, 2008). ^11^C-acetate also shows marked uptake in prostate cancer and has been shown to be more sensitive in detection of prostate cancer than ^18^FDG PET, but there are limited data on bone metastases (Oyama *et al*, 2002). Tracers targeted to prostate-specific membrane antigen and androgen receptor expression are also of increasing interest as response biomarkers (Apolo *et al*, 2008). However, as with many of the promising tracers for imaging metastatic prostate cancer, their role within clinical practice and clinical trials remains to be established within large prospective studies.

## Conclusion

**Figure 7:**
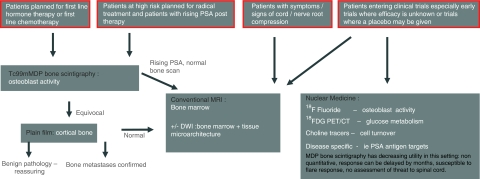
Flow chart showing decision pathways for imaging metastatic bone disease in patients with carcinoma of the prostate. Experimental/non-validated techniques are in italics.

In determining which diagnostic test should be used in the evaluation of bony metastases in prostate cancer, it is important to recognise that there are distinct patient groups for whom imaging objectives differ (Figure 7).

For patients embarking on radical therapy, the exclusion of bone metastases is paramount, making it imperative to use the most sensitive and specific diagnostic test available – currently this is MRI, which also evaluates potential threat to the spinal cord and nerve roots (evidence from prospective trials is summarised in Table 1). Future development may see MR imaging of the pelvis and whole spine as a baseline staging examination. Arguments that cost and availability limit its use may dwindle as the installed scanner base increases and faster imaging techniques are developed. As PET/CT procurement in the United Kingdom advances, ^18^F-fluoride PET/CT will challenge MRI as a diagnostic test for bone metastases in this setting, with its reported sensitivity and specificity of 100% (Even-Sapir *et al*, 2006). Further prospective studies comparing whole-body MRI and ^18^F-fluoride PET/CT, including cost analyses, should be encouraged.

For patients embarking on first-line hormone treatment or chemotherapy, bone scintigraphy in conjunction with PSA are adequate markers of bone disease status. In cases in which there are symptoms/signs of neurological compromise, MRI is of course the test of choice.

Early clinical trials require a robust and timely quantitative marker of disease response. In addition, given the extremely high incidence of metastatic bone disease in patients with carcinoma of the prostate entering clinical trials, impending cord compression should be identified with MRI and appropriate local treatment arranged if necessary. Further, the high sensitivity of MRI for lesion detection and capability for tumour dimension measurement can then be applied as an assessment of response to treatment (Tombal *et al*, 2005). Ultimately, the future may be with PET/MR – wide anatomical coverage and assessment of spinal cord and fusion of anatomical and quantitative data of tissue microarchitecture (DWI) with function from tracers such as choline or fluoride. At present, clinical trials demand a multiparametric approach with preliminary evidence, indicating that DWI should be pursued as a biomarker of metastatic bone disease. Alongside this, the development of specific targeted tracers such as choline or disease-specific targets such as PSA require investigation as they provide high sensitivity for disease activity.

In imaging prostatic bone metastases and their response to therapy, the heterogeneity of disease, patient selection bias and studies that lack a histopathological gold standard, make evaluation of the literature challenging. Further validation of imaging biomarkers of metastatic bone disease would do best if guided by consensus groups with the purpose of clearly defining objectives, prioritising imaging modalities to be taken forward, unifying ‘gold standards’ for bone disease and coordinating mulicentre prospective trials.

## Figures and Tables

**Table 1:** Detection of metastatic bone disease from carcinoma of the prostate: summary of prospective studies over the past 10 years

**Imaging modality**	**Structure/mechanism measured**	**Reference**	**Patients**	**Gold standard used**	**Sensitivity (%)**	**Specificity (%)**
Planar MDP bone scintigraphy	Osteoblast activity	Lecouvet *et al* (2007)	66	Consensus decision using bone scintigraphy, CT, MRI, follow-up, clinical and serum markers	46	32
		Even-Sapir *et al* (2006)	44	Consensus decision using ^18^F-fluoride PET/CT, and follow-up	69	64
SPECT		Even-Sapir *et al* (2006)	20	Consensus decision using ^18^F-fluoride PET/CT, and follow-up	92	82
MRI	Bone marrow	Lecouvet *et al* (2007)	66	Consensus decision using F/U bone scintigraphy, CT, MRI and clinical and serum markers	100	88
DW MRI	Bone marrow microstructure	Luboldt *et al* (2008)	11	^11^C-choline used as gold standard. In all, 15 true-positive bone metastases were identified by DWI, 15 by STIR and 14 true positives identified on T1W imaging	NA	NA
^18^FDG PET	Glucose metabolism	Morris *et al* (2002)	17	Consensus decision using bone scintigraphy. In all, 71% lesions visible on both modalities, 23% only on bone scan and 6% only on FDG PET	NA	NA
		Shreve *et al* (1996)	22	Consensus decision using bone scintigraphy and follow-up	65	
^18^F-fluoride PET/CT	Osteoblast activity	Even-Sapir *et al* (2006)	44	Consensus decision using bone scintigraphy and follow-up	100	100
		Beheshti *et al* (2008)	38	Consensus decision using ^18^F-fluorocholine PET/CT and follow-up	81	93
^18^F-fluorocholine PET/CT	Bone marrow – cellularity	Beheshti *et al* (2008)	38	Consensus decision using ^18^F-fluoride PET/CT and follow-up	99	85
^11^C-choline PET	Bone marrow – cellularity	Kotzerke *et al* (2000)	23	Consensus decision using bone scintigraphy. 11C-choline PET-matched bone scintigraphy for lesion detection	NA	NA

Abbreviations: CT= computerised tomography; DWI=diffusion-weighted imaging; MDP=methylene diphosphonate; MRI= magnetic resonance imaging; PET=positron emission tomography; SPECT=single-photon emission computerised tomography; STIR= short tau inversion recovery; SUV=standardised uptake value; ^18^FDG=^18^F-fluorodeoxyglucose.
